# Effectiveness and Safety of LMWH Treatment in Patients With Cancer Diagnosed With Non-High-Risk Venous Thromboembolism: Turkish Observational Study (TREBECA)

**DOI:** 10.1177/1076029617753538

**Published:** 2018-02-18

**Authors:** Ersin Ozaslan, Metin Ozkan, Irfan Cicin, Mustafa Benekli, Murat Kocer, Mukremin Uysal, Berna Oksuzoglu, Abdurrahman Isikdogan, Erdem Cubukcu, Emin T. Elkiran, Faysal Dane, Mehmet Aliustaoglu, Alper Sevinc, Aziz Karaoglu, Arife Ulas, Gamze Gokoz-Dogu

**Affiliations:** 1 Department of Medical Oncology, Erciyes University, Kayseri, Turkey; 2 Department of Medical Oncology, Trakya University, Edirne, Turkey; 3 Department of Medical Oncology, Gazi University, Ankara, Turkey; 4 Department of Medical Oncology, Suleyman Demirel University, Isparta, Turkey; 5 Department of Medical Oncology, Afyon Kocatepe University, Afyon, Turkey; 6 Department of Medical Oncology, Dr. Abdurrahman Yurtaslan Training and Research Hospital, Ankara, Turkey; 7 Department of Medical Oncology, Dicle University, Diyarbakir, Turkey; 8 Department of Medical Oncology, Ali Osman Sonmez Hospital, Bursa, Turkey; 9 Department of Medical Oncology, Inonu University, Malatya, Turkey; 10 Department of Medical Oncology, Marmara University Training and Research Hospital, Istanbul, Turkey; 11 Department of Medical Oncology, Dr. Lutfi Kirdar Kartal Education and Research Hospital, Istanbul, Turkey; 12 Department of Medical Oncology, Medical Park Gaziantep Hospital, Gaziantep, Turkey; 13 Department of Medical Oncology, Dokuz Eylul University, Izmir, Turkey; 14 Department of Medical Oncology, Yildirim Beyazit University Ataturk Training and Research Hospital, Ankara, Turkey; 15 Department of Medical Oncology, Pamukkale University Medical Faculty, Denizli, Turkey

**Keywords:** LMWH, VTE, bemiparin, enoxaparin, thrombosis, cancer

## Abstract

We compared the efficacy and safety of low-molecular-weight heparins (LMWHs) in patients with cancer who are at low risk of venous thromboembolism (VTE). Patients were treated by medical oncologists in Turkey at 15 sites, where they were enrolled and followed up for a period of 12 months. Due to the study design, there was no specific treatment protocol for LMWH. Primary end points were efficacy and the time to change in VTE status. Of the included 250 patients, 239 (95.6%), 176 (70.4%), 130 (52.0%), and 91 (36.4%) completed their day 15, month 3, month 6, and month 12 visits, respectively. Number of patients treated with enoxaparin, bemiparin, and tinzaparin were 133, 112, and 5, respectively. Anticoagulant therapy provoked thrombus resolution in 1.2% and 12.7% of patients using enoxaparin and bemiparin, respectively (*P* = .004). Thrombus resolution was observed in 81 more patients at month 3 visit. This ratio was 35 (40.2%) of 87 and 46 (54.1%) of 85 patients administered enoxaparin and bemiparin at the third visit, respectively (*P* = .038). Thrombus resolution was observed in 21 more patients during month 6 visit. This ratio was 5 (7.7%) of 65 and 15 (23.4%) of 64 patients administered enoxaparin and bemiparin at the fourth visit, respectively (*P* = .022). The LMWH was discontinued in only 2 patients due to gastrointestinal bleeding. This pioneering study shows bemiparin is more effective than enoxaparin in thrombosis resolution and has a similar tolerability profile.

## Introduction

Venous thromboembolism (VTE) is an important cause of death in patients with cancer. The estimated risk of VTE is 4 to 7 times higher among these patients compared to the normal population.^1,2^ Venous thromboembolism mostly occurs in the early months of chemotherapy.^1–3^ The incidence ranged from 8% to 19% depending on the tumor type. Tumors associated with the highest VTE risks are hematologic cancers followed by pancreatic, stomach, lung, ovarian, uterine, bladder, and brain tumors.^1^ A significantly higher proportion of VTE events was diagnosed in the outpatient setting compared to the inpatient setting. In a retrospective observational study in the United States, among 17.874 patients with cancer, 78% were outpatients and 22% were inpatients.^2^ Low-molecular-weight heparins (LMWHs), such as dalteparin, enoxaparin, tinzaparin, and bemiparin, are drugs of choice for VTE treatment and prevention because they enable outpatient treatment and eliminate the need for therapeutic monitoring in most patients.^4,5^ Few clinical studies have tested whether the clinical effects of these drugs are comparable.

Patients with cancer have a prothrombotic state resulting from the synergic activity of factors involved in the so-called Virchow’s triad. Stasis of the blood is caused by bed rest or the tumor compression; vascular injury is caused by intravasation of cancer cells, drugs, or therapeutic devices; and blood hypercoagulability is due to the release of cancer cell procoagulant factors, which affect the hemostasis process, including platelet functions and clotting cascade.^6^ As a consequence, interactions between cancer cell, host cell, and treatments activate the clotting process and cause various clinical presentations, such as abnormal laboratory results or massive thrombotic attacks.^7^

Treatment with LMWH is preferred compared to vitamin K antagonist (VKA) in patients with cancer.^5,8^ The effect of several LMWHs compared to VKA were evaluated in several randomized studies. In the ESFERA study of 583 patients, Santamaría et al assessed the clinical and economic outcomes associated with bemiparin versus VKA.^4^ In Meyer et al’s study of 146 patients with cancer, authors assessed whether a fixed dose of enoxaparin is superior to oral warfarin.^9^ In LITE study of 200 patients, assessing tinzaparin versus VKA, and CLOT study of 672 patients, assessing dalteparin versus coumarin, we showed LMWH was more effective and/or safer than VKAs in patients with cancer.^10,11^ However, if LMWH is not recommended, warfarin is an acceptable alternative for long-term therapy.^5,8^ Patients with cancer with deep vein thrombosis (DVT) or pulmonary embolism (PE) should be treated for a minimum duration of 3 months with either LMWH or warfarin. Low-molecular-weight heparin is recommended for the first 6 months of chronic treatment of proximal DVT or PE.^5^ And CHEST VTE treatment guideline recommended extended anticoagulant therapy over 3 months of therapy in patients with DVT or PE and active cancer.^8^ Anticoagulation for an indefinite duration should be considered in patients with active cancer. Since the chronic treatment of VTE with LMWH has not been evaluated in clinical trials of patients with cancer longer than 6 months, the decision to continue LMWH beyond 6 months or to switch to warfarin therapy should be based on clinical judgment.^5,12^

In the present observational study, we compared the effectiveness and safety of available LMWHs (enoxaparin, bemiparin, or tinzaparin) selected based on clinician’s judgment in outpatients with cancer who had low probability risk of developing VTE.

## Methods

### Study Population

Patients with cancer meeting all of the following criteria were included in the study: patients who were 18 years or older, with a signed informed consent, who have been diagnosed for VTE, and who has a minimum life expectancy of 6 months. Patients meeting any of the following criteria were excluded from the study: patients with active bleeding or at risk of bleeding; patients who had major surgery in the last 7 days; patients who were at high risk of PE; patients with cardiopulmonary instability, severe systemic venous occlusion, and thrombocytopenia (<50 000/µL); inpatients under medical or surgical supervision; patients with low ability to communicate and for whom it is not possible to provide care at home; patients with an INR ≥1.5 due to liver dysfunction, diagnosed for cerebral vascular aneurism, active gastric, and/or duodenal ulcer, diagnosed for bacterial endocarditis, severe renal dysfunction, unstabilized hypertension, <35 kg or ≥110 kg of weight; and patients who are allergic to heparin and who have a history of cerebrovascular event in the last 1 month.

Life-threatening cardiopulmonary instability, which is also considered as an exclusion criterion and required hospitalization, was defined as high-risk VTE. Patients not required hospitalization because of VTE were defined as low-risk VTE and included in the study.

### Consent

Written informed consent was obtained from all patients for participation in the study after a review of the protocol, their responsibilities, and their rights. Consent was also obtained for recording of their data and collection, as outlined in the protocol, to allow regulatory monitoring, statistical analysis, and peer review presentation and publication of the study results.

### Study Design

TREBECA is a multicenter, noninterventional, prospective, and observational study assessing the effectiveness and safety of long-term LMWH for the treatment of VTE in low-risk patients with cancer and registered in the Clinicaltrials.gov with the registration number NCT02017743, and the last time verification was on December 2013. The study was conducted in 15 centers all around Turkey. Treatment choice was done according to the physician’s clinical judgment. Treatment of the patients with cancer diagnosed with VTE was recorded. Patients’ recruitment period was 8 months, and patients were followed up for a period of 12 months.

### Treatment and Follow-Up

The exact dose of LMWH administered subcutaneously once or twice daily is based on the dosage scheme according to the patient’s body weight. Patients are given labeled kits containing syringes of LMWH containing 4000 IU, 6000 IU, or 8000 IU of enoxaparin twice daily; 5000 IU, 7500 IU, or 10000 IU of bemiparin once daily; and 10 000 IU, 14 000 IU, or 18 000 IU of tinzaparin once daily, whichever dose is most appropriate for their weight.

Venous thromboembolism was diagnosed depending on the clinical and radiological evaluation. Eastern Cooperative Oncology Group performance status (ECOG PS), a commonly used scale in patients with cancer, which ranges from 0 (PS: 0, being fully functional and asymptomatic) to 4 (PS: 4, being bedridden), was evaluated clinically, laboratory tests were performed, and concomitant diseases, treatments and adverse events (AEs), if any, were evaluated and recorded. Radiological assessments that were carried out were Doppler ultrasonography, spiral thorax computer tomography (CT), and CT venography.

Patients were monitored during the follow-up period of 12 months with the following 5 visits: first evaluation visit (day 0 visit), day 15 visit, month 3 visit, month 6 visit, and finally month 12 visit. In each visit, ECOG PS, concomitant medications and LMWH-associated AEs, d-dimer, and blood counts (eg, hemoglobin, platelets) of patients were evaluated. Furthermore, Doppler ultrasound of extremities, diametrical difference in extremities, and physical examination of patients with DVT were carried out. Spiral thorax CT and pulmonary examination were carried out for patients with pulmonary thromboembolism. Computed tomography venography was also performed in rare cases.

The LMWH was given for at least 3 months and was continued if the patient did not show thrombus resolution or even if thrombosis was resolved and the patient still had high risk factors for thrombosis. Temporary discontinuation of LMWH therapy, if not exceeding 2 weeks, was permitted in case of thrombocytopenia (platelet count less than 50 × 10^9^/L) or bleeding events or if the patient had to undergo any invasive procedure. If study drug was held or missed for more than 2 consecutive weeks, then the patient was considered to have permanently discontinued study drug.

### The Primary Objectives of the Study

The primary objective of the study was the evaluation of the effectiveness of LMWH used in the treatment of thrombosis in patients with cancer having VTE, including early-stage thrombus regression (clinical and/or radiological assessment) and VTE recurrence at a later stage (rethrombosis rate). Additionally, we assessed the safety of LMWH used in the treatment of thrombosis in patients with cancer having VTE and thus compared different LMWH treatments in terms of effectiveness and safety in the treatment of VTE in patients with cancer (based on the rate of thrombosis regression and presence/absence of rethrombosis).

### The Secondary Objectives of the Study

Incidence of VTE, factors that affect VTE occurrence, cancer type, and VTE site breakdown in patients with cancer having VTE, and observation of patient compliance to LMWH treatment in outpatients were established as secondary objectives of the study.

## Results

### Patient Characteristics

Data for 250 patients who met the study inclusion criteria were examined and analyzed. Of them, 239 (95.6%) patients completed their day 15 visit, 176 (70.4%) completed their month 3 visit, 130 (52.0%) completed their month 6 visit, and 91 (36.4%) completed the entire study. One hundred thirty-three patients were treated with enoxaparin, 112 patients were treated with bemiparin, and 5 patients were treated with tinzaparin. The reasons and numbers of patients who discontinued the therapy during the follow-up period were as follows: 111 patients died, 34 patients were lost to follow-up, 8 patients failed to give informed consent for follow-up, and 2 patients had serious AEs (eg, gastrointestinal bleeding).

The mean age of the patients was 60.2 ± 13.7, while 133 (53.2%) of the patients were women. Colorectal (21.2%), lung (16.8%), and breast (14.8%) cancers were the most common forms of cancer. Among these patients, 200 (80%) patients had ECOG PS 0 and 1. One hundred thirty-four patients were never smokers, and smoking history was not available for 44 patients. Patient characteristics are presented in Table 1.

**Table 1.** Patient Characteristics.

Mean age	60.2 ± 13.7 years
Sex ratio F:M	133 (53.2%):117 (46.8%)
Cancer types, n (%)	
Colorectal	53 (21.2)
Lung	42 (16.8)
Breast	37 (14.8)
Gynecological	25 (10)
Urological	24 (9.6)
Gastric	22 (8.8)
Pancreas	19 (7.6)
Other	28 (11.2)
Smoking history, n (%)	
Ex smoker	49 (19.6)
Current smoker	23 (9.2)
Nonsmoker	134 (53.6)
ECOG performance status (PS), n (%)	
PS 0	67 (26.8)
PS 1	133 (53.2)
PS 2	50 (20)

Abbreviations: ECOG, Eastern Cooperative Oncology Group; F, female; M, male.

It was determined that 173 (69.2%) patients were metastatic at the baseline visit, 80% (n = 200) had previous or ongoing chemotherapy, and 73 (29.2%) patients had previous or ongoing radiotherapy. Twenty-five (10%) patients had central catheter and 40 (16%) patients were not mobilized when thrombosis occurred, and only 10 patients had a history of thrombophilia.

Thromboembolism was detected in the lower extremities in 66.8% of the patients, in 14.8% in the upper extremities, and in 19.2% in the lung, and 8 of the patients had thrombus on the other sites. The occurrence of thrombosis in the left lower extremity was 1.7 times more compared to the right lower extremity. The occurrence of thrombosis in the right lung was 2.87 times more compared to the left lung. There was no significant difference between the upper extremities. The most common sites of thrombosis were lower extremities in all malignancies (*P* < .05). The site of tumor and the high occurrence of thrombosis were as follows: the rate of thrombosis in the lower extremity was most common among gynecological cancers (88%), whereas the rate of thrombosis in the upper extremity was most common in breast cancers (27.1%). The rate of thrombosis in the lungs was most common in lung cancers (26.2%; Table 2).

**Table 2.** Rates of Thrombus Location According to Primary Tumor Sites.

Primary Tumor Sites	n	Thrombus Location, %		
		Lower Limb	Upper Limb	Lung
Lung	42	64.2	11.9	26.2
Breast	37	51.3	27.1	21.6
Colorectal	53	75.4	15.1	11.3
Stomach	22	59.1	22.7	22.7
Pancreas	19	63.1	15.8	10.5
Urological	24	62.5	16.7	20.8
Gynecological	25	88.0	0	12.0
Others	28	67.9	7.1	25.0

### Effectiveness of LMWH

**Figure 1.**
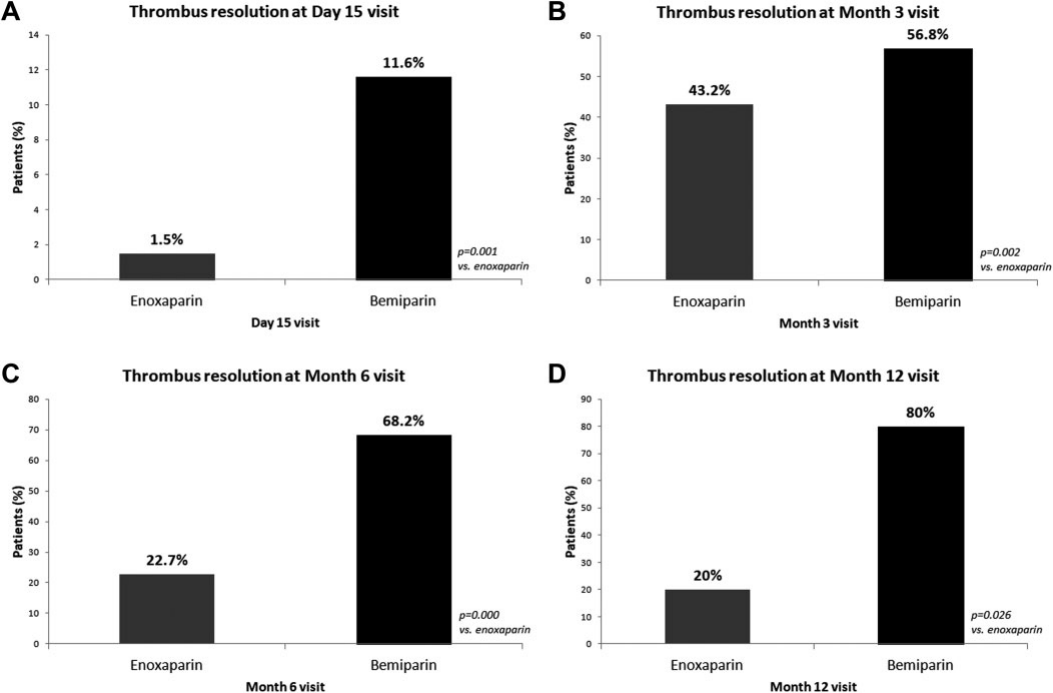
A, Impact of anticoagulant therapy in the thrombus resolution in the early stages of treatment. B, Impact of anticoagulant therapy in the thrombus resolution at month 3 visit. C, Impact of anticoagulant therapy in thrombus resolution at month 6 visit. D, Impact of anticoagulant therapy in thrombus resolution at month 12 visit.

**Figure 2.**
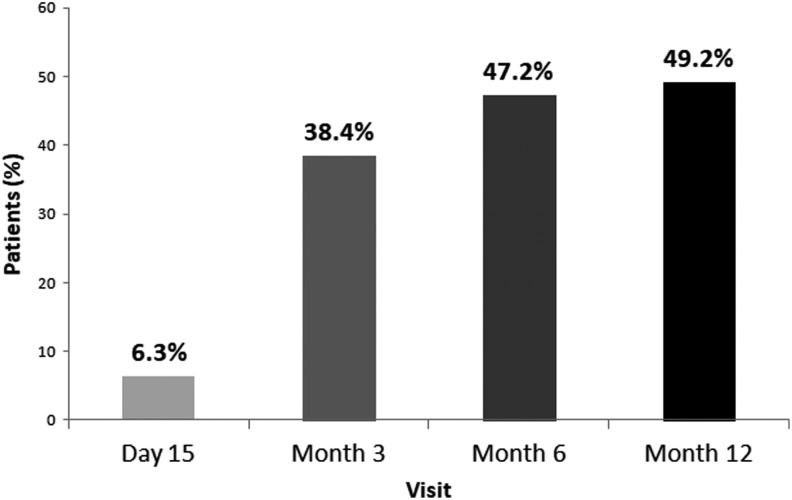
Thrombus resolution with low-molecular weight heparin (LMWH) treatment during follow-up period.

Anticoagulant therapy provoked thrombus resolution in 15 (6.3%) of the patients on day 15. The thrombosis was dissolved in 1.5% of the patients using enoxaparin and in 11.6% of the patients using bemiparin (*P* = .001; Figure 1A). The LMWH was replaced or the dose of the current drug was increased in cases where no reductions were observed in the thrombus. Thrombosis was dissolved in 81 more patients (46%) by month 3 visit. This ratio was 35 (43.2%) of 81 among patients taking enoxaparin and 46 (56.8%) of 81 among patients taking bemiparin at the time of the third visit (*P* = .002; Figure 1B). Thrombosis was dissolved in 22 more patients (16.9%) by month 6 visit. This ratio was 5 (22.7%) of 22 among patients taking enoxaparin and 15 (68.2%) of 22 among patients taking bemiparin at the time of the fourth visit (*P* = .000; Figure 1C). Thrombosis was dissolved in 5 (5.5%) more patients by month 12 visit. This ratio was 1 (20%) of 5 among patients taking enoxaparin and 4 (80%) of 5 among patients taking bemiparin at the time of the 12th month visit (*P* = .026; Figure 1D). Among 5 patients receiving tinzaparin, thrombus was resolved in 2 patients in month 6 visit and 4 other patients discontinued therapy during the follow-up period. The total rates for the thrombus resolution during the 3-month and 6-month period were 38.4% and 47.2%, respectively (Figure 2).

Allocation of LMWH treatment based on primary tumor sites is presented in Table 3. When we evaluated the relationship between primary tumor sites and thrombus resolution, rates of thrombus resolution in breast, lung, gastric, and gynecological cancers were much higher compared to the rates of thrombus resolution in colorectal, pancreas, and urological cancers (Table 4). Thrombus recurrence was observed in 10 patients during the follow-up period after LMWH treatment was discontinued. Total rate of recurrence was 4.1%.

**Table 3.** Allocation of LMWH Treatment Based on Primary Tumor Sites Is Presented.

Primary Tumor Sites	n	Received LMWH, %	
		Enoxaparin	Bemiparin
Lung	42	45.2	54.8
Breast	37	59.5	40.5
Colorectal	53	50.9	49.1
Stomach	22	54.5	45.5
Pancreas	19	57.9	26.3
Urological	24	66.7	33.3
Gynecological	25	44.0	52.0
Others	28	53.6	42.9

**Table 4.** Rates of Thrombus Resolution in Evaluated Patients According to Primary Tumor Sites.

Primary Tumor Sites	Day 15	Month 3	Month 6	Month 12
Lung	7.1% (3/42)	61.3% (19/31)	5.5% (1/18)	0% (0/9)
Breast	10.8% (4/37)	53.8% (14/26)	22.7% (5/22)	11.8 (2/17)
Colorectal	3.8% (2/52)	34% (16/47)	13.5% (5/37)	8.3% (2/24)
Stomach	10% (2/20)	66.7% (10/15)	11.1% (1/9)	0% (0/5)
Pancreas	0% (0/17)	33.3% (2/6)	NE	NE
Urological	0% (0/22)	35.3% (6/17)	15.4% (2/13)	0% (0/11)
Gynecological	8.7% (2/23)	58.8% (10/17)	14.3% (2/14)	10% (1/10)

Abbreviation: NE, not evaluated.

### Safety of LMWH

Low-molecular-weight heparin was discontinued in only 2 patients due to gastrointestinal bleeding; aside from this, there were no other serious drug-related AEs requiring discontinuation. Both patients who had gastrointestinal bleeding had gastric cancer. One of these major bleedings occurred in a patient under bemiparin treatment and the other bleeding event was fatal while on enoxaparin treatment (Table 5). There were no patients with grade 3 or 4 thrombocytopenia or other AEs.

**Table 5.** LMWH Adverse Events.

	Bemiparin	Enoxaparin
Major bleeding	1 (0.89%)	1 (0.75%)
Minor bleeding	0 (0.0%)	1 (0.75%)

Abbreviation: LMWH, low-molecular-weight heparin.

## Discussion

Cancer and VTE are closely related. Indeed, cancer can reveal VTE and VTE can be the first sign of cancer. Low-molecular-weight heparin is now the first-line treatment in patients with cancer.

Types of tumor associated with the highest VTE risk are pancreatic, stomach, lung, ovarian, uterine, bladder, and brain tumors.^2^ In the IMPACT study, 27 479 patients with cancer had received chemotherapy and patients with a history of VTE within 12 months during chemotherapy were evaluated. Rates of VTE according to primary tumor sites were pancreas, 21.3%; stomach, 16.2%; lung, 14.8%; colorectal, 11.7%; ovary, 11.4%; and bladder, 9.8%.^3^ However, most common primary tumor sites are colorectal, lung, and breast in our study. The reason of why the VTE ratio was lower in patients with pancreatic cancer and stomach cancer could be the consequence of the low number of patients.

Randomized cohort studies showed that LMWHs are more effective, are safer, have positive contribution on survival, do not need monitoring, and drug interactions with chemotherapeutics are less.^4,8–15^ Therefore, compared to VKA, LMWHs are the treatments of choice in patients with cancer. Low-molecular-weight heparins such as dalteparin, enoxaparin, tinzaparin, and bemiparin are drugs of choice for VTE treatment and prevention, since they enable outpatient treatment and eliminate the need for therapeutic monitoring in most patients. Few clinical studies have tested whether the clinical effects of these agents are comparable. In the literature, studies usually compared LMWHs with VKAs. On the other hand, in our study, LMWHs are compared to each other where there is lack of data.

For long-term anticoagulation, LMWH is preferred for at least 6 months. Anticoagulation with LMWH beyond the initial 6 months may be considered for selected patients with active cancer such as those with metastatic disease or those receiving chemotherapy.^5^ It is recommended that all patients should be reassessed in 5 to 7 days to ensure symptomatic improvement after starting LMWH.^16,17^ Data on the frequency of patient evaluation who are given LMWH for the treatment of VTE as well as the dose increments and the switch time of LMWH were not provided. In our study, our first evaluation was on day 15, clinically and radiologically. Clinical and radiological resolution of the thrombus was statistically significantly higher in patients receiving bemiparin compared to patients receiving enoxaparin, on day 15, month 3, and month 6 visits. This could result from the differentiated pharmacological profile of bemiparin compared to other LMWHs: Bemiparin has a lower molecular weight than enoxaparin (3.600 vs 4.500 Da), and even it has the lowest molecular weight among all currently marketed LMWHs.^18^ In addition to that, what determines the effectiveness of LMWH is antithrombin activity, thus anti-factor-Xa activity.^19^ Previous published studies have shown that the anti-factor Xa–anti-factor IIa ratio of enoxaparin is between 3.3 and 5.3, whereas this ratio is 8.0 for bemiparin.^18^ As this ratio increases, effectiveness/safety correlation of LMWH might increase as well. In a study of Borrell et al, the prophylactic doses of bemiparin and enoxaparin have been compared and the serum anti-factor-Xa activity has been found to be higher; likewise, the duration of serum anti-factor-Xa activity has been found longer for bemiparin.^20^ The longer mean half-life of 5.3 hours for bemiparin compared to 4.3 hours for enoxaparin is another fact for bemiparin being more efficacious than enoxaparin.^18^ The longer half-life provides a longer duration of the drug in the blood, thus providing the effective dose for a longer time. The bioavailability of the route of subcutaneous administration of LMWH is also different. The bioavailability of bemiparin is 96% compared to 92% of enoxaparin.^21^ It might be concluded that lower molecular weight, longer half-life, higher anti-factor-Xa activity, and higher bioavailability of bemiparin make it more effective in the thrombus resolution in our study.

In many previous studies, LMWHs have been proved to be safer than warfarin. When we evaluated related studies, Meyer et al showed that prolonged treatment with enoxaparin is safer than with warfarin in patients with cancer.^9^ And the ESFERA study indicated that bemiparin is a safer and cost-neutral alternative to warfarin for long-term treatment of VTE.^4^ In the ESFERA study, major bleeding rate was only 0.4% in bemiparin group and 1.7% in warfarin group. In our study, we also observed that major bleeding rates were very low in patients receiving both bemiparin and enoxaparin.

Although the present research has reached its aims, there were some limitations. First, it was not a randomized study, but an observational study, and because of the multicenter design, radiological imaging of the patients, the principle indicator of the thrombus resolution, was carried out by the various radiologists. Due to disease progression, some of the patients did not respond to cancer therapies, and therefore, they did not respond to LMWH either. Since no information is available, of which LMWH was administered to these treatment-resistant patients, this can be considered as a limitation of the present observational study.

## Conclusion

In conclusion, this is the first comprehensive study comparing LMWHs, head to head, in patients with cancer having VTE. The observation that bemiparin is more effective in resolution of thrombosis was noteworthy. It was observed that thrombosis could not be effectively treated within the first 15 days in a significant portion of patients, but it can be concluded that the effectiveness of the treatment increases after month 3. Therefore, we can say that a treatment of at least 3 months is appropriate for patients with cancer, even among those with a low risk of VTE.
